# Exploring Sexting and Online Sexual Victimization during the COVID-19 Pandemic Lockdown

**DOI:** 10.3390/ijerph18126662

**Published:** 2021-06-21

**Authors:** Aina M. Gassó, Katrin Mueller-Johnson, José R. Agustina, Esperanza L. Gómez-Durán

**Affiliations:** 1 School of Law, Universitat Internacional de Catalunya, 08017 Barcelona, Spain; 2 Centre for Criminology, Oxford University, Oxford OX1 2JD, UK; Katrin.mueller-johnson@crim.ox.ac.uk; 3 School of Law, Universitat Abat Oliba CEU, 08017 Barcelona, Spain; jagustinas@uao.es; 4 School of Medicine, Universitat Internacional de Catalunya, 08017 Barcelona, Spain; elgomez@uic.es

**Keywords:** sexting, online sexual victimization, image-based sexual abuse, lockdown, COVID-19

## Abstract

The COVID-19 pandemic lockdown has impacted daily routines, forcing people to stop socializing in person and changing the way people express their feelings and their romantic or sexual interactions. Social distancing has changed the way people behave online, and we expect that engagement in sexting and online sexual victimization behaviors have increased during lockdown. The aim of this paper is to study the prevalence of sexting and online sexual victimization behaviors during the COVID-19 lockdown in Spanish adults in order to explore how social distancing has affected these behaviors. The sample comprised 293 Spanish adults (mean age = 30.3; 66.2% female) who took part in an online survey about their engagement in sexting behaviors and online sexual victimization experiences. Overall results were apparently not supportive of our main hypothesis, showing that both sexting engagement and online sexual victimization decreased during lockdown despite the increase in internet use. Apart from differences in time period of reference, some alternative hypotheses relate to the increased presence of capable guardians according to the routine activities theory and to forced distance as a demotivation to sext. Possible explanations and hypotheses for these results are discussed further in the paper.

## 1. Introduction

The COVID-19 pandemic lockdown has impacted daily routines in many ways. It forced people to stop socializing in person and non-cohabiting couples to spend weeks apart, and it has changed the way people express their feelings and their romantic or sexual interactions. As a result, there have been media reports on the increase of sexting and also, worryingly, of sexual harassment and victimization using phones or online media [1,2,3]. Although controversial, sexting has been defined as the act of sending, receiving, and/or forwarding nude or semi-nude pictures or videos through electronic devices and/or social media platforms [4,5]. Voluntary and consensual sexting between adults is considered by most scholars to be a form of normal sexual expression [6]; however, it has also been conceptualized as a risky behavior that increases vulnerability to online sexual victimization (OSV), such as sexting coercion, non-consensual dissemination of sexual content, revenge porn, sextortion, or online grooming [7]. Psychological consequences of OSV range from victims’ low self-esteem, decrease of academic performance, and sleep alterations to more severe implications, such as depression, anxiety, and suicidal ideation, and many suicide cases related to OSV have been publicized widely in the media [8].

From a psychological perspective, such so-called exceptional events, like the COVID-19 pandemic, act as natural experiments because they change the structure of routine activities that can reorganize how offenders, victims, and capable guardians converge and interact in time and space [9]. The rapid development of the COVID-19 pandemic forced the Spanish government to implement strict quarantine measures, imposing social distancing and limiting social contact and human interaction, which increased the frequency of internet use [10]. Recent reports have highlighted that internet use increased by 74%, and social media use increased by 55% during the COVID-19 lockdown in Spanish population [10]. Previous data about sexting and OSV are available for the Spanish population, but to date, there is scarce data regarding the effects of the lockdown or social distancing on sexting or online sexual victimization behaviors in Spain. Previous literature states that sexting is a common form of sexual interaction, through the Internet, between heterogeneous people and within heterogeneous relationships [1,7,11]. It has also been reported that an increase in the frequency of internet use is strongly correlated with an increase in sexting engagement [11]. Furthermore, during the pandemic, sex with people outside the household posed a significant risk of SARS-CoV-2 transmission, so interpersonal sexual contact at a distance could be solved by technology-mediated sexual interactions [12,13]. In line with these findings, Ballester–Arnal et al. reported that almost 50% of their adult Spanish sample increased their online sexual activities during the COVID-19 lockdown [14]. In fact, during this forced social quarantine, media campaigns encouraged people to perform cybersex and sexting to maintain what they called sexual sanity, but they sometimes neglected to inform of the possible risky outcomes, such as online sexual victimization [12]. Although it depends highly on many various contextual factors, participation in sexting increases the probability of reporting OSV, especially when the addressee is a stranger [8,11]. Therefore, the more people sext, the greater the risk that they may be sexually victimized online [11]. Thus, we expect sexting engagement to have increased during lockdown, and, subsequently, we expect to see an increase in OSV. 

The aim of this paper is to analyze the prevalence of sexting and online sexual victimization behaviors during the COVID-19 lockdown in a convenience sample of Spanish adults in order to explore how social distancing affected these behaviors. This paper’s main aim is to quantitatively measure how the current situation and short-term future of social distancing and lack of human contact is affecting sexting engagement and OSV in the Spanish population. Compared to previous data about sexting and OSV, we expected that the disruption of routine activities and psychosocial factors would have increased sexting and OSV prevalence rates, with all of the social, legal, and psychological consequences that derive from it.

## 2. Materials and Methods

### 2.1. Participants

The sample comprised 293 Spanish adults, including 194 women (66.2%) and 96 men (32.8%) and 3 participants (1%) who did not specify their sex. Ages ranged from 18 to 73 years old, with a mean age of 30.34 years (SD = 13.02). The descriptive statistics for the demographic variables for the total sample can be found in Table 1.

### 2.2. Procedure

The study was approved by the Ethics Committee of the International University of Catalunya (UIC Barcelona), with approval code DRET-2020-01. Data were collected using an online survey. Participants were recruited via social media platforms such as Twitter, LinkedIn, WhatsApp, and Instagram and mail lists and self-selected to take part on their own time without any compensation. Participation was voluntary and responses were anonymous to promote openness and honesty. Informed consent was obtained explicitly by asking the participant to tick a consent box prior to starting out on the survey. The questionnaire took approximately 20–25 min to complete, and once completed, participants were given information on community resources they could access in case of distress and for victim support. The investigator’s email address was provided to contact in case of concerns, but no participant contacted the investigator. Response rate at the time of data analysis was 59%. 

### 2.3. Measures

For the purpose of this research, we defined sexting as creating, sending, and/or forwarding nude or sexually explicit images or videos through any electronic device. Sexting coercion was defined as the use of coercive tactics to solicit sexually explicit photos and videos from someone; sexting coercion victimization was defined as the act of being pressured, threatened, and/or blackmailed by someone to send them sexual content and/or being a victim of non-consensual dissemination of the shared sexual content.

#### 2.3.1. Sexting Questionnaire

To measure consensual sexting and online sexual victimization, we used five sexting items adapted from the Juvenile Online Victimization Questionnaire (JOV-Q [15]) to assess different types of online sexual victimization behaviors. For the purpose of this research, the selected variables were (1) voluntary sexting, which was assessed using the following question: “During lockdown, I have created and sent to someone else photos or videos with sexual content of myself.”; and (2) online sexual victimization, which comprised the four following dichotomous items:

1. “During lockdown, someone has shared, disseminated, or posted online my nude or sexual photos/videos without my consent.”
2. “During lockdown, someone has pressured (repeatedly insisted) me to send them photos or videos of my sexual content.”
3. “During lockdown, someone has threatened me to send them photos or videos of my sexual content.”
4. “During lockdown, someone has blackmailed me to send them my sexual photos/videos of myself.”

Furthermore, participants were asked about the frequency of each of the measured behaviors and who they had engaged in the behavior with.

#### 2.3.2. Socio-Demographic and Background Questionnaire

We included questions about age, sex, educational attainment, marital status, parental marital status, place of residence, living situation during lockdown, employment situation during lockdown, and questions about frequency of use of phones and social media.

### 2.4. Data Analysis

For the purpose of this research, we conducted statistical analysis using SPSS 25. Prevalence rates were calculated using cross-tables, and statistical differences between groups were calculated using chi-square measures, setting statistical significance at *p =* 0.005. 

## 3. Results

### 3.1. Prevalence of Sexting and Online Sexual Victimization 

Out of 293 participants, almost 30% created and sent their own sexual imagery during the COVID-19 lockdown (27.1% of males and 30.4% of females), without significant differences between males and females (χ|^2^ (1, *n* = 290) = 0.344, *p* = 0.558, OR = 0.85, 95% CI (0.49, 1.46)). Up to the age of 50 years, the prevalence of sexting was similarly high, with 34.4% among 18–30-year-olds, 26.2% among 31–40-year-olds, and 27.6% among 41–50-year olds. Among the over 50-year-olds, then, sexting prevalence was only 3.3%. Regarding frequency of voluntary sexting, results showed that, out of the total number of participants who reported engaging in voluntary sexting, 5% sexted only once, 12% sexted two or three times throughout lockdown, 5% sexted once or twice a month, 5% sexted once or twice a week, and 2% sexted almost every day. Sexting also differed by relationship status. Only 7.1% of married respondents sexted, while those in a relationship, divorced/or separated, and singles all sexted at higher rates (36.9%, 25.0%, and 30.0%, respectively).

Nearly 10% of the sample had experienced OSV during this time (9.7%), with significantly higher rates for women (12.4%) than for men (4.2%) (χ|^2^ (1, *n* = 290) = 4.96, *p* = 0.026, OR females = 3.25, 95% CI (1.09, 9.64)). Singles had the highest rate of victimization (15.0%), followed by those in a relationship (7.4%) and respondents who were married (2.4%) (in this sample, no divorced person experienced OSV). In particular, it was singles aged 30 or younger that had an elevated rate (17.7% compared to 4.2% of singles aged 31 years and above.). Single women aged 30 or less had the highest rate of 21.5% OSV compared to 9.7% of single men aged 30 or younger. Regarding frequency of the online sexual victimization received, one participant (0.3%) reported being a victim of non-consensual dissemination of sexting once during lockdown. Furthermore, 7% of participants reported being pressured to sext, with the following frequency: 1.7% (*n* = 5) reported being pressured once, 4.4% (*n* = 13) reported being pressured 2–3 times, 1.3% (*n* = 4) reported being pressured once or twice a month, and one participant (0.3%) reported being pressured once or twice a week during lockdown. Only one participant (0.3%) had been threated to sext, with a frequency of 2–3 times during lockdown, and two participants reported being blackmailed to sext (0.6%), with one of them being blackmailed once during lockdown and the other 2–3 times during lockdown. 

Voluntary sexting and OSV were significantly associated (|χ^2^ (1, *n* = 293) = 31.11, *p* < 0.000, OR = 9.23, 95% CI (3.75, 22.7)), with participants who sexted being 9.23 times more likely to be sexually victimized online than those who did not sext. However, there was no significant association between frequency of sexting and likelihood of victimization (z = −0.094, *p* = 0.925). These results indicate that sexting engagement is associated with being online sexually victimized, but that the frequency of engagement in sexting is not a relevant variable in this association.

### 3.2. Sender and Receiver of the Sexting Content

When examining results regarding who participants had sexted with or been victimized by, results showed that out of participants who sexted voluntarily, 7.4% did so with a friend, 18.9% with a partner, 3.4% with an ex-partner, 5.1% with an internet acquaintance, and 0.7% with a stranger. For those who had been victimized, results differed by behavior. The participant who reported being a victim of non-consensual dissemination reported being victimized by an ex-partner. For being pressured to sext, 3% were pressured by a friend, 2% by a partner, 0.3% by an ex-partner, 4% by an internet acquaintance, and 1.3% by a stranger. Furthermore, the person who reported being threatened to sext was threatened by an internet acquaintance (0.3%), and out of those who reported being blackmailed to sext, 0.7% were victimized by a friend, 0.3% by an internet acquaintance, and 0.7% by a stranger. 

### 3.3. Living Situation during COVID-19, Sexting, and OSV

Results indicate that participants’ living situation during the COVID-19 pandemic was significantly related to voluntary sexting (χ|^2^ (1, *n* = 293) = 16.06, *p* = 0.007). Participants who lived by themselves sexted the most (44%), followed by those who lived with friends or in a shared flat (40%) and by those who lived with their parents (33.8%). Those who lived with their partners (15.4%) and those who lived with their partner and children sexted the least (12.8%).

With regards to OSV, results indicate that the living situation during the COVID-19 pandemic was not significantly associated with an increase in victimization (χ|^2^ (1, *n* = 293) = 9.76, *p* = 0.082). However, there was a marginal overrepresentation of people who lived with friends among those who were victimized (standardized residual = 2.1).

### 3.4. Internet Use during COVID-19, Sexting, and OSV

Finally, results regarding internet use during the COVID-19 pandemic showed that there is an association between the time people spent online and engagement in voluntary sexting (z = 2.82, *p* = 0.005). However, results showed that more time spent online was not significantly related to online sexual victimization (χ|^2^ (1, *n* = 293) = 4.29, *p* = 0.368), indicating that OSV is not associated with spending more time online.

## 4. Discussion

The aim of this study was to analyze the prevalence of sexting and OSV behaviors during the COVID-19 lockdown in a convenient, exploratory sample of Spanish adults in order to explore how the lack of social contact has affected these behaviors. The main hypothesis was that a change in routine activities, such as an increase in the use of the Internet, would be associated with an increase in sexting engagement and, subsequently, an increase in OSV. Our results support the hypothesis only partially.

Overall, our results showed a sexting prevalence rate of almost 30% for the 3-month period of lockdown, whilst reported rates before lockdown range from 47% to 82.2% yearly prevalence [7,16,17,18,19]. Consistently, a recent Australian study on sexuality during lockdown measured different variables amongst a sample of 965 adults and reported that only 30% of their adult sample had engaged in sexting during the COVID-19 lockdown [20]. Similarly, previous literature also reported general online sexual victimization yearly prevalence rates around 30% in adult samples before social distancing, whilst the OSV prevalence rate was almost 10% during the 3-month period of lockdown [7,8].

We believe these results could be explained by different hypotheses. First of all, the data gathered for the present study asked participants to report sexting engagement and OSV during the COVID-19 lockdown, which comprised a shorter period of time (three months—from mid-March to mid-June) than most studies, which look at yearly prevalence or lifetime prevalence [7,8,16]. It is also important to acknowledge the relatively small sample of this preliminary report on sexting during COVID. Differences in prevalence rates may be due to differences in the sample composition, such as, for instance, in age structure. Furthermore, our sample is composed primarily of those who are young, students, and living at home, which are sociodemographic factors that have been pointed out by the literature in relation with the engagement in voluntary sexting and the risk of OSV [7,21]. 

However, several factors may have influenced sexting and OSV rates during lockdown. Firstly, the routine activities theory could explain a change in behavior trends by relating behavior to everyday social interaction [22]. Our data empirically supports a positive relationship between sexting and opportunity. Similarly to previous literature, our results showed that there is an association between living alone and sexting engagement and that those who lived with a third party (friends, parents, partner) sexted less than those who lived by themselves, which is in line with other research [13,23]. The presence of the so-called capable guardians might discourage engagement in sexually-related activities, such as voluntary sexting, and might also discourage the perpetration of OSV [24]. The lack of social contact during lockdown changed the routine activities of one or all (capable guardians but also potential sexters or potential victims of OSV), which changed the likelihood of convergence in space and time, ultimately changing the likelihood of sexting and OSV occurring. On the other hand, it is also possible that people who lived by themselves found easier opportunities to sext or were more likely to find themselves in the mood and/or more inclined to seek this form of social connection than people who were spending lockdown in the presence of others [13].

Finally, engagement in sexting and thus OSV might not be increasing during lockdown because, despite the technological component of the behaviors, people might feel more inclined to share their intimate sexual content with others they have previously met, and social distancing reduces the possibility of acquiring new social bonds [14]. Thus, forced physical distance may be demotivation to sext. Our results show a prevalence of sexting among those who were not living with a partner ranging from 33.8% to 44%, whilst only 12.8–15.4% of those who were living with a partner sexted. In pre-COVID circumstances, Currin, Pascarella, and Hubach [25] found that a sizeable minority of their sample (50) identified a non-sex-related reason of physical distance from their partner(s) as a motivation to sext (comforting, anxiety reduction, and/or distance); however, the majority (86) of respondents identified sex-related reasons for engaging in sexting. Literature has also reported that individuals in romantic relationships are more likely to sext and that only 26% of those in committed relationships reported “Sexting due to their partner being far away” as a motivation to sext [16,25,26,27]. Furthermore, a recent study analyzed changes in sexual behaviors during the pandemic lockdown in a sample of 965 Australian adults and found a significant decrease in the use of dating apps, which could partially account for a decrease in sexting engagement with unknown people [20]. As participation in sexting increases the probability of OSV, especially when the addressee is a stranger, sexting primarily within relationships could also contribute to a lower rate of OSV [25,26,27,28]. 

## 5. Conclusions

Our hypothesis was partially supported because results indicate that internet use is associated with sexting engagement, as has been reported by previous literature [21,29]. However, OSV was not associated with internet use. A possible explanation for these results might be that the relationship between OSV and internet use is mediated by engagement in sexting, considering results showing a positive correlation between sexting engagement and OSV. On the other hand, these results might be explained by the fact that people are using the Internet in a healthier way, and despite spending more time online, they do it more safely. 

Since sexting and OSV are social phenomena, changes in their characteristics and prevalence rates should be expected when social context dramatically changes, as it happened during the 2020 lockdown. Further research is needed specifically addressing these potential determinants of sexting and OSV in order to develop accurate and effective prevention strategies. 

### Limitations and Future Research

This study has several limitations that should be considered. First, this study is cross-sectional, so no causal relationships can be established between the variables. Second, the reliability of the measures was not tested due to the sample size. Third, the sample used was a convenient and self-selected via an online survey, with a high percentage of young students and participants living at home. The ratio of males to females is not even, and other instances of bias in the sample selection might affect the results, so extrapolation of the data should be cautiously done. It would be important that other studies replicate these results with larger sample sizes and other samples (such as adolescents and college students). Further research should try to compare results from pre-COVID-19 samples to actual samples in order to identify possible changes in behavioral trends.

## Figures and Tables

**Table 1:** Descriptive statistics of demographic and background variables for the sample.

	% (*n* = 293)	Mean (SD)	Min	Max
**Demographic Variables**				
**Gender**				
Male	32.8			
Female	66.2			
**Age**		30.3 (13.0)	18	73
**Sexual Orientation**				
Heterosexual	83.6			
Homosexual	5.1			
Bisexual	10.2			
Other	1.0			
**Civil Status**				
Single	41.0			
In relationship	41.6			
Married	14.3			
Divorced/separated	2.7			
**Education Level**				
Secondary education	2.7			
Postsecondary education	40.6			
Vocational qualification	8.9			
Undergraduate	25.3			
Postgraduate	21.2			
Doctorate (PhD)	1.4			
**Living Situation During COVID-19**				
Alone	8.9			
Shared flat	4.1			
Living with partner	18.8			
Living with partner and kids	13.0			
With parents	52.2			
Other	5.70			
**Employment Status During COVID-19**				
Student	43.0			
Unemployed before COVID-19	9.2			
Unemployed because of COVID-19	6.1			
Unemployed but doing child care	2.0			
Home office	21.8			
Essential services	11.3			
Other	6.5			
**Health Situation During COVID-19**				
Hospitalized due to COVID-19	0			
Home isolation	1.7			
COVID-19 disease light/moderate	0.3			
Other	1.7			
**Own Smartphone**	98			
**Frequency of Internet Access During COVID-19**				
Once a week	2.7			
2–3 times a week	2.0			
Everyday less than 1 h	19.5			
2–3 h per day	35.5			
More than 3 h per day	40.3			
**Increased Use of Internet During COVID**				
Yes	85.7			
**Social Media Use During Lockdown**				
Yes	95.6			
No	4.1			

## Data Availability

The data used to support the findings of this study are available from the corresponding author upon request.
